# Analogy of Irian Uveitis in Man, with Dry Periodic Fluxion in the Horse

**Published:** 1898-02

**Authors:** 


### FRENCH.

UNDER THE DIRECTION OF DR. ALEXANDER GLASS,
PHILADELPHIA, PA.

ANALOGY OF IRIAN UVEITIS IN MAN, WITH DRY PERIODIC FLUXION IN THE HORSE.—Dr. Rolland has carefully studied and determined the nature of the disease in the horse called periodic fluxion—periodic ophthalmia—because it is considered by most veterinarians as almost incurable and leading finally to blindness. Previous to Roland's study of this disease, veterinarians had a very vague conception of the nature and the seat of this affection.

The author has clearly proved that in the horse there exist two varieties of periodic ophthalmia.

1. A true inflammatory ophthalmia, occurring in successive attacks and complicated with a hypopyon and even hypohema (pus and blood in the anterior chamber), which is analogous to rheumatismal iritis in man ; the latter is also often complicated with hypopyon and even hypohema when the inflammatory process extends to the ciliary body.

2. A dry periodic fluxion, without any very apparent symptoms of irritation other than posterior synechia of the pupil. This is analogous to irian uveitis.

Rolland recommends a very simple and rapid means of detecting periodic fluxion in the horse. It consists in instilling into the eye of the horse a few drops of a solution of atropin. If the animal is subject to dry ophthalmia, and more especially, if it is inflammatory, pupillar adhesions of the iris (posterior synechia) can be detected at the end of about ten minutes.

I have dilated the pupils of a number of horses brought to the clinic of the Lyons Veterinary School for diseases other than of the eye. In two days I have found two cases of periodic fluxion ; one horse, aged eight years, was without doubt affected with irian uveitis, or dry periodic fluxion ; the other, four years old, was somewhat doubtful. The owners of these two horses said that they had never observed anything abnormal in the eyes of these animals ; they had only noticed that both horses, previously kind and gentle, had for several months been shying and showing fear of objects, so much so as to become unsafe to drive. With my knowledge of the functional symptoms of irian uveitis in man, this change in an animal's disposition can be explained without difficulty; these horses, seeing before their eyes black points, and at times cobweb-like structures somewhat phosphorescent, imagine that they are external objects and endeavor to avoid them.

Rolland believes that periodic fluxion of the eye of the horse is hereditary, and that such animals should not be used for breeding. I believe that this opinion is well founded, especially with reference to inflammatory periodic fluxion; that is to say, iritis or irido-choroiditis, which, being generally of a rheumatoid nature, should evidently transmit itself by heredity. However, I am of the opinion, from numerous observations upon irian uveitis, or dry periodic fluxion in man, that dry periodic fluxion in the horse is not hereditary, or at least has no well-defined tendency to be so

However it may be, not being satisfied merely with the diagnosis of dry periodic fluxion in the horse with atropin, as indicated by Rolland, I have radically cured it by iridectomy, which has succeeded so well in man. I practised this operation in the two cases indicated above, and in one case the periodic fluxion was not doubtful. It is very evident that, as the animal in question ceased to shy and became manageable, iridectomy cures dry periodic fluxion in the horse as it does in the human species. We will even extend this treatment to inflammatory periodic fluxion if atropin, one of its curative agents, becomes ineffectual.—Dr. Grandclement, in *Ann. de Méd. Vét.*

